# Evaluation of youth male soccer players' injuries in the context of body posture

**DOI:** 10.1002/jeo2.70334

**Published:** 2025-07-13

**Authors:** Dorottya Szabó, Gabriella Kiss, Eva Tékus, Judit Diana Fekete, Csaba Vermes, Tibor Mintál

**Affiliations:** ^1^ Medical School, Sports Medicine Center University of Pécs Pécs Hungary; ^2^ Department of Languages for Biomedical Purposes and Communication, Medical School University of Pécs Pécs Hungary; ^3^ Department of Orthopaedics Medical School University of Pécs Pécs Hungary

**Keywords:** football, injury, posture, youth

## Abstract

**Purpose:**

The number of injuries among junior footballers is extremely high. Adolescent footballers may be at particularly high risk due the changes during fast growth period. Their poor posture could be in association with incidence of injuries. Our aim was investigate the relationship between poor posture and injury characteristics of youth football players.

**Methods:**

One hundred and sixteen football players (*n* = 116, age range 11–18 years) were studied. We grouped our subjects by age group and academic group. A questionnaire and injury registration software were used to assess training and injury‐related data. Habitual posture was analysed using the PostureScreen mobile application.

**Results:**

Injury rates were high in the overall study population, typically highest in the U14 and U15 age groups. Significant positive correlations were found between several postural parameters and injury characteristics. Less significant correlations were observed according to academic grouping.

**Conclusions:**

There is a clear relationship between postural parameters measured in the frontal and sagittal planes and injury incidence in youth football players.

**Level of Evidence:**

Level III.

**Abbreviations**
BMI: body mass index
HA: head angulation
HT: head translation
KA: knee angulation
KT: knee translation
PA: pelvis angulation
PT: pelvis translation
SA: shoulder girdle angulation
ST: shoulder girdle translation
TFAA: total frontal anterior angulation
TFAT: total frontal anterior translation
TFDA: total frontal dorsal angulation
TFDT: total frontal dorsal translation
TSLA: total sagittal left angulation
TSLT: total sagittal left translation
TSRA: total sagittal right angulation
TSRT: total sagittal right translation

## INTRODUCTION

Football is one of the most popular sports worldwide in terms of the number of athletes, with millions of players participating at all levels of competition (adult, junior, competitive and recreational) [15, 18, 21, 27]. The football academies incorporate, help athletic development of youth footballers, where the U15–U19 age group is actually considered an academy athlete and the younger age groups (U12–U14) are classified as preacademic players [36]. Football has a high injury rate, not only because of its contact nature, but also because of the sport‐specific asymmetric load (e.g., using mostly dominant leg for shots) [2, 5, 8, 13, 19, 20, 25, 50]. Taking into account the joint and muscle strain, football is an acyclic activity that involves a lot of jumping, landing, sprinting and changing direction, which also carries a risk of injury [33]. The volume and intensity variations could differ in soccer training in a wide range between players (e.g., total distance per training session: 4584.5–7062.66 m, distance on 20 km/h speed per trainings: 27.42–29.42 m and numbers of sprints 0.05–3.5) [10]. Consequently, the players have to show a high level of speed, endurance, strength and agility.

For youth players, the level of these skills is in correlation with changes during growth such as development of poor body posture [1, 2]. The rate of growth plays a significant, potential role in the damage caused, in addition, adolescent football players are at particularly high risk of injury due to the combined effect of the demands of football‐exertion, while simultaneously undergoing significant biomechanical changes associated with relatively rapid growth [36]. This period of peak growth is associated with physiological changes [34] during which soft tissues may be subjected to increased stress and strain [13, 23], which can temporarily affect motor skills and performance so that functional deficits can develop [2]. Existing literature suggests that the risk of injury in adolescent footballers is greatest during this rapid growth and remains elevated for up to 6 months afterwards [27, 35, 36]. Moreover, according to Kemper et al., if the monthly increase in height exceeds 0.6 cm, the likelihood of injury can rise by 1.63 times [27].

The term ‘postural dysfunction’ or ‘poor posture’ is used to describe a condition where the sagittal curvatures of the spine deviate from their normal physiological alignment but can be corrected by active muscular activity indicating mobility. It is worth noting that sagittal deviations in posture often coincide with deviations in the frontal plane of the spine [28]. Adolescents frequently encounter the issue of poor posture, a condition that has been extensively discussed in various scientific articles [13]. Various studies have shown the effects of sports training and football on body posture and found positive and negative aspects [2, 3, 14]. In the case of footballers, the variation of pelvic area is typically characterised by alteration [19]. In this sport, evidence suggests that poor posture can pose a risk of injuries [4, 9, 50]. Studies on footballers have shown that the incidence of injuries is highest among 13–14‐year‐olds, especially in the U14–U15 age group [54]. A significant proportion (78%) of these injuries affect the lower limb, and around 45%–72% fall into the category of noncontact injuries. The most commonly observed noncontact injuries in football include soft tissue complaints and hamstring muscle injuries [44, 54]. Inadequate fitness levels, previous injuries and muscular imbalances are frequently identified in the literature as potential risk factors for sustaining such injuries. Poor posture, including trunk and pelvic asymmetries as well as sagittal spinal deviations, may contribute to an increased risk of lower limb injuries, even though it is not the primary cause [18, 19, 43, 52]. These facts highlight the importance of postural assessment and follow‐up in the prevention of noncontact injuries in football.

Postural assessment is a key component of sports medicine testing. Traditional methods like observation and goniometry have long been used, while advancements in digital technology have introduced techniques such as photogrammetry and Moiré topography [29, 30, 44]. Radiography remains the gold standard, particularly with EOS 3D microdose scanning [30, 44]. The PostureScreen mobile app has also been validated for reliability in postural analysis [6, 46, 47].

Coaches and player care professionals implement preventive programs to reduce posture‐related injuries in adolescent footballers [32, 39, 40, 42]. Enhancing posture helps minimise injury risk, boost performance and ensure a safer sporting experience [2].

Despite the importance of posture in injury, research on its relationship with injuries in adolescent footballers is limited. Existing studies mainly focus on sagittal plane abnormalities, with little exploration of frontal plane deviations and their impact on injuries, highlighting the need for further investigation.

However, comprehensive growth and injury data are scarce and the existing literature on this subject is lacking. Furthermore, there is a shortage of studies in literature examining the relationship between postural abnormalities and injury occurrence in adolescent footballers. Additionally, current research focuses primarily on sagittal plane abnormalities in relation to posture, neglecting to investigate frontal plane abnormalities and their potential impact on injuries, which requires further exploration. We hypothesised that occurrence and recurrence of injuries is related to different body posture parameters measured by PostureScreen application.

The aim of our study was to shed light on possible associations between injuries and postural parameters, specifically in the sagittal and frontal planes, in young football players considering specified age groups. Studies on these young individuals may contribute to the theoretical understanding of sports physiotherapy and sports medicine, in addition to their practical and sporting benefits, furthermore they could provide valuable support for athletes' long‐term sporting goals, their daily activities and even their participation in lifelong physical activities.

## MATERIALS AND METHODS

### Participants and protocols

A cross‐sectional survey was carried out using convenience sampling methods. Participants were aged between 11 and 18 years old, who had at least 4 years of experience in football and engaged in at least four training sessions per week (with an average of 6 h per week dedicated to football training). At the time of the investigation, the academy only had male athletes, why boys were included in this study. Exclusion criterion was the absence of informed consent. The study was carried out among youth players of a national football talent centre.

A total of 116 football players were surveyed. These players were divided into eight groups based on chronological age (U12–U19) and two academic groups (preacademic: from U12 to U14, academic: from U15 to U19). Academic classification is a general used categorisation of players age at football academies. To determine the body composition of the participants, the bioimpedance analyzer InBody 270 (In‐Body 270, Biospace) was utilised. Additionally, accurate height measurements were obtained using the portable anthropometer (Seca 213 stadiometer, Seca Corporation) for all participants. To evaluate the age and training frequency of the athletes, we employed a self‐edited questionnaire. Injury data were obtained from the TalentX (Done IT Innovation Ltd.) injury registration database, which is utilised by the sports club participating in our study. The injuries were diagnosed by the team doctor (traumatologist, orthopaedist) and recorded the injuries sustained during football activities. Injuries were classified based on various parameters, including the type of injury (acute injury and overload injury). The overload injury was defined as the onset an injury without a single, identifiable event [17]. The injuries were categorised as follows: Injuries to joints, injuries to different muscle groups (front of thigh, back of thigh, inner thigh, calf, inguinal region, gluteal region and trunk), specific subtype of injury (fracture, ligamentous injury, sprain, muscle injury, pain, inflammation, osteochondritis and cartilage injury), side of the body and region of the body affected. We also observed recurrent injuries, which defined as the same type of injury at the same site which occurs after a player's return to full participation in trainings [17]. The severity of injuries is determined by the number of training days missed by the player [17]. We collected data on injuries occurring within the year preceding the study (July 2022–June 2023 involved total preparation and competition period with a 3‐week‐long winter break).

Our study was approved by the Regional and Institutional Committee of Science and Research Ethics of our university (permit number: 7939‐PTE 2019, date of approval: 08.06.2019) and it was conducted in accordance with the principles of the Declaration of Helsinki.

### Examination of habitual standing posture

**Figure 1:**
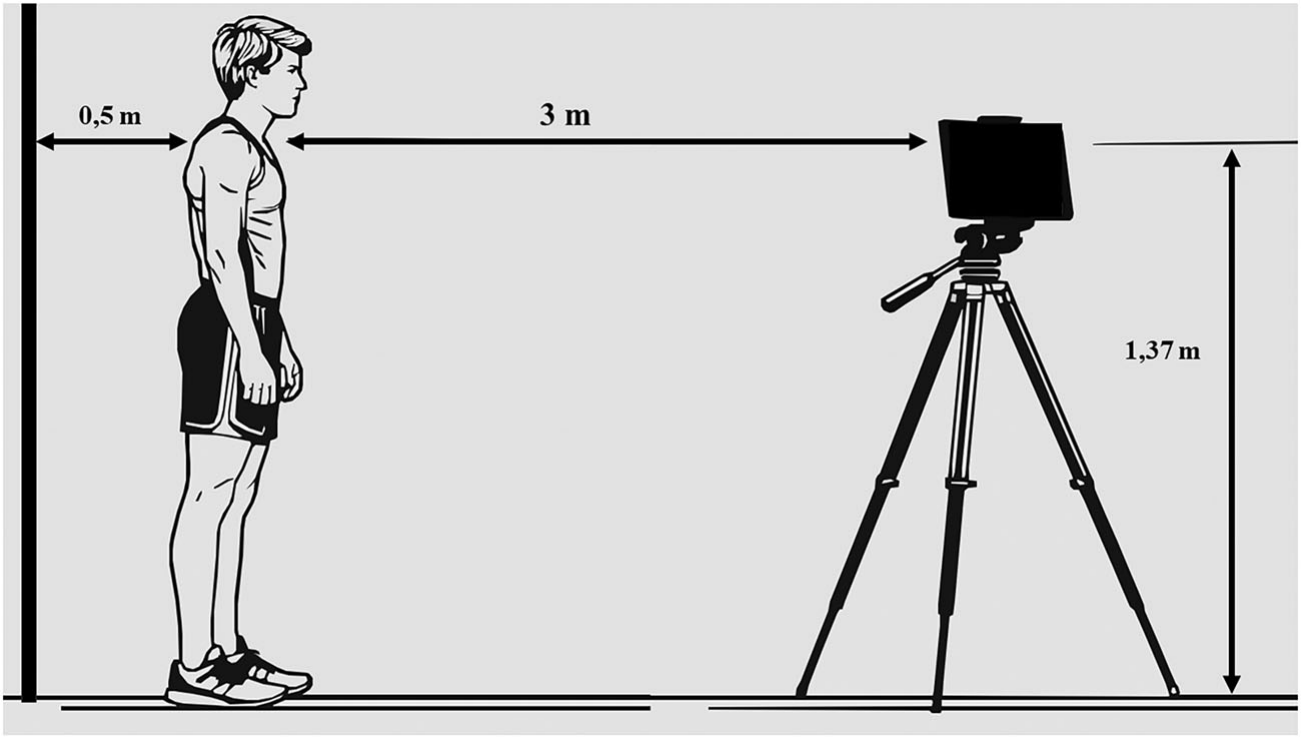
Capturing method of body posture.

The postural analysis of the athletes was conducted using the Posture Screen mobile application (PostureCo, Inc.; Szucs et al.) [6, 46, 47] by two measurers (experienced physiotherapist) from each subject. According to Boland's method [6] to conduct the assessment, four images were captured for each athlete: one frontal view in the frontal plane, a rear view and two side views in the sagittal plane. To ensure consistency, the position of the subject and camera was set at a standardised distance and height (Figure 1). To enhance visibility and accuracy, it was necessary for the athletes to be barefoot and to wear only underwear when taking pictures [6].

The analysis of the habitual posture was automatically performed by the application after marking the required orientation points. Throughout the process, the software evaluated the posture by assigning a ‘total’ score to all four sides being examined, which is the cumulative deviation of the selected body parts (head, shoulders, ribcage, pelvis and knees) from the optimal, central axis running from ear to malleolus lateralis in lateral view and in the frontal view divides the body into two equal parts. The deviations known as ‘total’ encompass both displacements, referred to as translation (T), and as angulation (A). The measurement for translation is expressed in centimeters, while angulation is measured in degrees [46].

**Figure 2:**
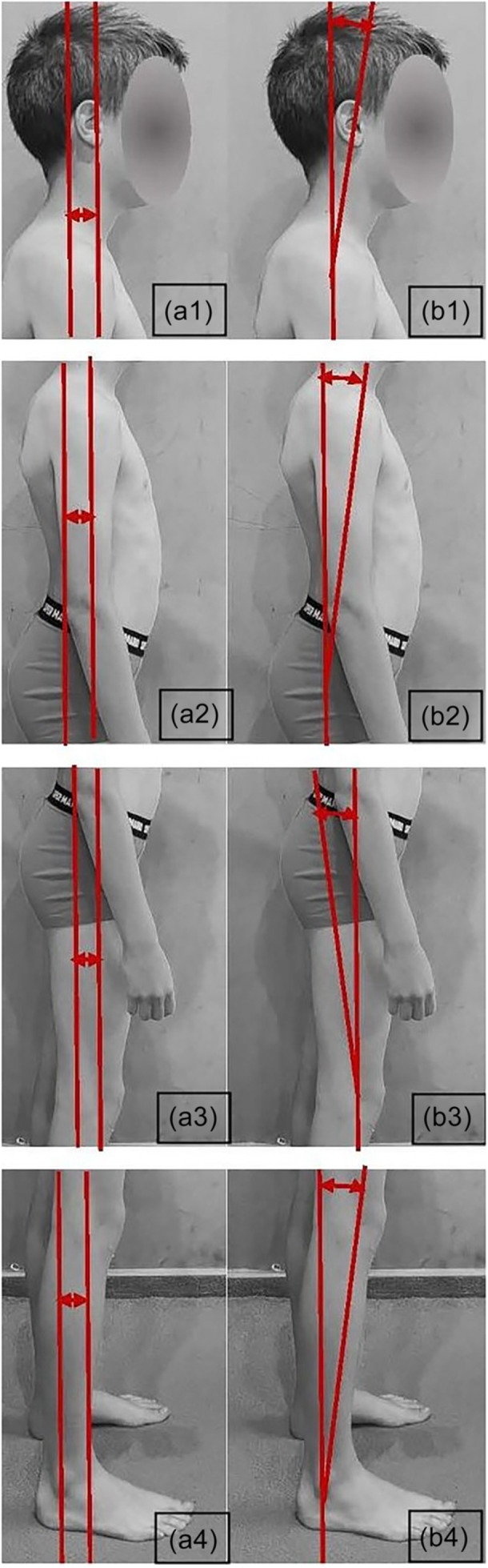
Method of calculating the translation (a1: head translation, a2: shoulder translation, a3: hip/pelvis translation and a4: knee translation) and angulation (b1: head angulation, b2: shoulder angulation, b3: hip/pelvis angulation and b4: knee angulation) of each body part in right aspect.

The assessment includes detailed data on the translation and angulation deviations of specific body parts, such as the head, shoulder belt, pelvis and knee, from the optimal alignment. For the optimal body posture, the translation and angulation data are close to 0. The recorded values for these parameters are as follows. The variables measured in this study include (Figure 2):

1. Head translation (HT—a1 in Figure 2) and head angulation (HA—b1 in Figure 2).
2. Shoulder girdle translation (ST—a2 in Figure 2) and shoulder girdle angulation (SA—b1 in Figure 2).
3. Ribcage translation (RT) and ribcage angulation (RA).
4. Pelvis translation (PT—a3 in Figure 2) and pelvis angulation (PA—b1 in Figure 2).
5. Knee translation (KT—a4 in Figure 2) and knee angulation (KA—b1 in Figure 2).

The following variables are defined by the application using digitised anatomical landmarks (Table 1) [41].

1. Total frontal anterior translation (TFAT) and total frontal dorsal translation (TFDT): represent the cumulative displacement of different body parts from the optimal line in the frontal plane from frontal and posterior views.
2. Total sagittal right translation (TSRT) and total sagittal left translation (TSLT): refer to the cumulative displacement of different body parts from the optimal line in the sagittal plane from right and left side views.
3. Total frontal anterior angulation (TFAA) and total frontal dorsal angulation (TFDA): refer to the cumulative deviation of different body parts from the optimal line in the frontal plane in frontal and dorsal views.
4. Total sagittal right angulation (TSRA) and total sagittal left angulation (TSLA): represent the cumulative deviation of the different body parts from the optimal line in the sagittal plane in right and left side views.

**Table 1:** Counting method of total parameters.

Region of the body	Frontal translation	Frontal angulation
Head (H)	X	X
Shoulder (S)	X	X
Ribcage (R)	X	X
Hip/pelvis (P)	X	X
Knee (K)	n/a	n/a
Total	Total frontal anterior translation	Total frontal anterior angulation

Region of the body	Dorsal translation	Dorsal angulation
Head (H)	X	X
Shoulder (S)	X	X
Ribcage (R)	X	X
Hip/pelvis (P)	X	X
Knee (K)	n/a	n/a
Total	Total dorsal anterior translation	Total dorsal anterior angulation

Region of the body	Sagittal translation	Sagittal angulation
Head (H)	X	X
Shoulder (S)	X	X
Ribcage (R)	X	X
Hip/pelvis (P)	X	X
Knee (K)	n/a	n/a
Total	Total sagittal right/left translation	Total sagittal right/left angulation

Abbreviation: n/a, nonapplicable.

### Statistical analysis

The analysis was performed using SPSS 28.0 (IBM). Descriptive statistics were computed for each variable. The groups were categorised based on chronological age and academic classification. According to academic classification, football players were divided into two groups (preacademic: between U12 and U14, academic: between U15 and U19) on the basis of the chronological age. According to the results of the Kolmogorov–Smirnov test, the differences between seven age groups (in postural parameters) were calculated with one‐way analysis of variance (ANOVA) with Tukey's multiple comparisons test (for normal distribution) and Kruskal–Wallis *H*‐test with pairwise comparisons (for nonnormal distribution). In addition, an independent samples *T*‐test was used to examine differences in postural parameters between academic classification groups (for normal distribution).

A two‐way mixed‐effects consistency model was used for the intertester reliability analysis of the PostureScreen data. The coefficients of the PostureScreen variables were between 0.7 and 0.97, the 95% confidence interval (CI) −0.29 to 0.58. To qualify the effect sizes Hedges' *g* values were calculated based on the significantly changed variables and *d* values were interpreted based on Hedges' method [24].

Based on the mean values of postural parameters, two distinct groups were established: one above the mean and one below the mean. The relationship between presence and recurrence of injury and categorical variables was analysed within the specific groups and across the entire sample using chi‐square test. We used discriminant analysis to build a predictive model for the potential risk of muscle injury depending on postural parameters. The significance level was *p* < 0.05. In figures and tables, mean ± standard deviation data are reported consistently.

## RESULTS

### Anthropometric results

Participants' anthropometric indicators and years of football training are shown in Table 2.

**Table 2:** Anthropometric data and training years.

	Age		Weight		Height		BMI		Years of football training	
Age groups	Mean (years)	SD (±)	Mean (kg)	SD (±)	Mean (cm)	SD (±)	Mean (kg/m^2^)	SD (±)	Mean (years)	SD (±)
U12 (*n* = 9)	11.1	0.33	39.7	7.50	149	6.72	17.9	2.34	5.60	1.00
U13 (*n* = 14)	12.0	0	42.7	7.00	156	8.76	17.4	1.39	6.78	0.80
U14 (*n* = 13)	13.0	0	53.5	9.58	166	8.38	19.2	2.00	6.69	0.94
U15 (*n* = 17)	13.9	0.24	58.3	10.90	174	9.68	19.2	1.96	7.88	0.85
U16 (*n* = 24)	14.8	0.38	62.2	7.34	174	7.31	20.6	2.31	9.12	1.26
U17 (*n* = 20)	16.3	0.44	65.3	10.30	176	6.57	21.0	2.30	9.95	1.23
U19 (*n* = 19)	17.8	0.53	71.0	7.46	178	6.70	22.3	1.38	11.10	1.32

Abbreviations: BMI, body mass index; SD, standard deviation.

### Incidence of injuries by age group

**Figure 3:**
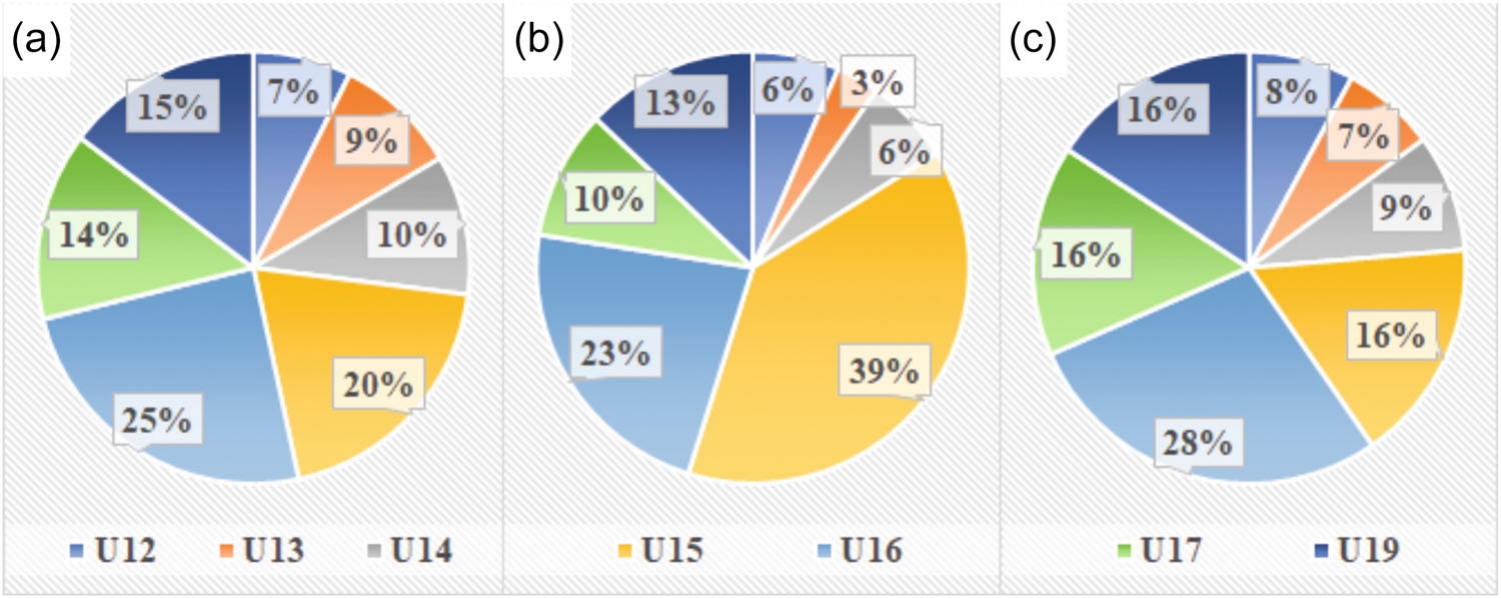
Distribution of injuries and injury types by chronological age groups (U12–U19). (a) Injury incidence in age groups. (b) Acute injury incidence in age groups. (c) Overload injury incidence in age groups.

The results were evaluated and 84.4% of the 116 subjects reported injury. As there was more than one injury per individual, this gave a total of 145 injuries. The distribution of sports injuries and different sports injuries by age group is shown in Figure 3. All injuries that did not involve acute structural damage to tissues were included in the overload injuries.

**Figure 4:**
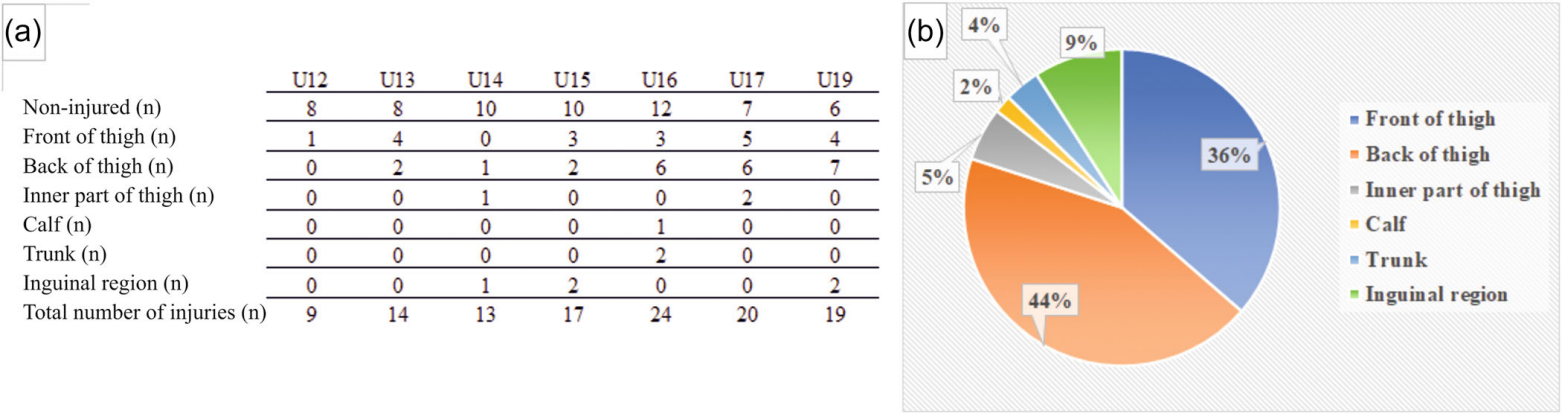
Distribution of muscle injuries. (a) Distribution of muscle injuries by age groups. (b) Distribution of muscle injuries by body parts.

Of the total injuries, 91% involved the lower limb. Among the injury subtypes we identified, 70.6% of athletes suffered a muscle injury (Figure 3), mostly affected (80%) parts were the front and back of the thigh. Furthermore, the frequency (13.8%) of the chronic tuberosity tibiae or heel pain was reported. The distribution of the most common muscle injuries by age group is shown in Figure 4.

### Postural parameters

Most of the injuries mentioned above (54.1%) required missing 1–3 weeks of training, 27.6% 4–6 weeks, 7.1% 7–9 weeks and 8.2% more than 10 weeks. Looking at all injuries, 44.1% had a recurrence of the same injury over the 1‐year study period.

**Figure 5:**
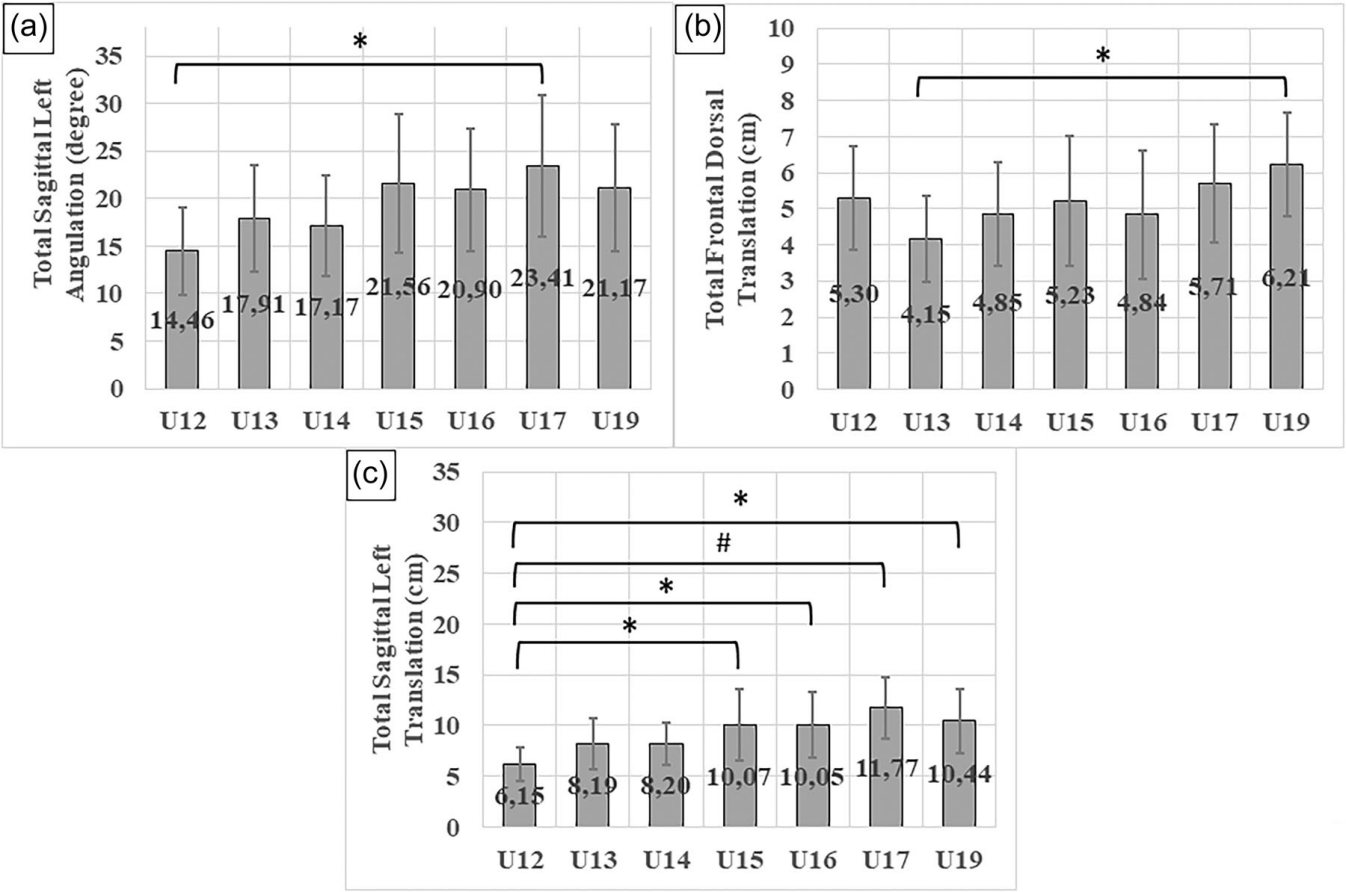
Differences in postural parameters between age groups. (a) Total sagittal left angulation (TSLA; Hedges's *g* 1.33 and 95% confidence interval [IC] 0.475–2.191). (b) Total frontal dorsal translation (TFDT; Hedges's *g* 1.536 and 95% IC 0.753–2.32). (c) Total sagittal left translation (TSLT; Hedges's *g* 1.284 and 95% IC 0.404–2.164, Hedges's *g* 1.345 and 95% IC 0.513–2.177, Hedges's *g* 2.089 and 95% IC 1.137–3.042, Hedges's *g* 1.54 and 95% IC 0.65–2.43), *p < 0.05, ^#^ *p* < 0.001.

Applying one‐way ANOVA with Tukey post hoc test, posture analysis found significant differences between chronological age groups in three (TSLA, TFDT and TSLT) of the eight ‘total’ parameters (TFAT, TFDT, TSRT, TSLT, TFAA, TFDA, TSRA and TSLA) (Figure 5).

Using one‐way ANOVA with Tukey post hoc test, significant differences in pelvic translation (PT) were observed when comparing different age groups, specifically between U13–U17 (*p* = 0.005; Hedges's *g* 1.416; 95% IC 0.655–2.178), U13–U19 (*p* = 0.015; Hedges's *g* 1.786; 95% IC 1.222–2.35), U14–U17 (*p* = 0.009; Hedges's *g* 1.15; 95% IC 0.414–1.886) and U14–U19 (*p* = 0.027; Hedges's *g* 1.293; 95% IC 0.535–2.05). The most substantial difference was found between the mean values of U13 and U17 (*p* = 0.005). Additionally, there was a notable difference in pelvic angulation (PA) among the age groups. The value for U13 was significantly lower than that of U17 (*p* = 0.011; Hedges's *g* 1.364; 95% IC 0.608–2.12) and U19 (*p* = 0.043; Hedges's *g* 1.621; 95% IC 0.341) age groups. Furthermore, the measurement of HT exhibited a significant disparity between the U12 and U19 age groups (*p* = 0.033; Hedges's *g* 1.193; 0.341–2.046) used Kruskal–Wallis test, with the U19 value being greater.

The subjects were categorised into two distinct groups based on their academic age, specifically preacademy U12–U14 and academy U15–U19. Significant differences were observed between these two groups in terms of the average scores for TFAT (*p* = 0.009; Hedges's *g* −0.2; 95% IC −0.594 to 0.194), TSRT (*p* = 0.004; Hedges's *g* 0.455; 95% IC 0.057–0.852), TSLT (*p* = 0.004; Hedges's *g* 0.769; 95% IC 0.363–1.174), HT (*p* < 0.001; Hedges's *g* 0.938; 95% IC 0.527–1.35) and HA (*p* = 0.014; Hedges's *g* 0.719; 95% IC 0.315–1.123) based on the results of independent sample *T*‐test.

### Relationships between postural parameters and injury variables

First, the postural parameters were calculated and averaged according to age groups. Subsequently, these averages were categorised as either above or below the overall average. The relationship between injury categories (presence and recurrence of injuries) and categorical variables (above the mean and below the mean), as determined by the postural parameters, was analysed within the specific groups and also across the entire sample.

Applying chi‐square test, the U13 age group exhibited the strongest associations between postural parameters and injury characteristics. Across various age groups, irrespective of grouping, the occurrence and nature of injuries were primarily influenced by the overall frontal plane deviations of the total postural parameters (TFAA, TFDT and TFDA), as well as greater deviations of the pelvis (PT). These findings are summarised in Table 3.

**Table 3:** Correlations between injuries and postural parameters (A: chronological age groups, B: academic groups and total sample as SUM), *r*: Cramer V coefficient, level of significance *p* < 0.05.

(A) Age groups	Injury yes/no	Repetitive injury	(B) Age groups	Injury yes/no	Repetitive injury
U13 (*n* = 15)	TFDT		Preacademic (*n* = 44)	KA	TFDA
	*p* = 0.02 *r* = 0.6			*p* = 0.042 *r* = 0.306	*p* = 0.05 *r* = 0.361
	TFDA	TFDA			PT
	*p* = 0.05 *r* = 0.492	*p* = 0.043 *r* = 0.648			*p* = 0.022 *r* = 0.461
	PT	PT	Academic (*n* = 119)	PT	TFAT
	*p* = 0.003 *r* = 0.764	*p* = 0.011 *r* = 0.773		*p* = 0.05 *r* = 0.225	*p* < 0.001 *r* = 0.368
	TSLT	TSLT		PA	PA
	*p* = 0.013 *r* = 0.604	*p* = 0.035 *r* = 0.627		*p* = 0.011 *r* = 0.232	*p* = 0.039 *r* = 0.234
		KA	SUM (*n* = 163)		TFAT
		*p* = 0.05 *r* = 0.591			*p* = 0.004 *r* = 0.263
U15 (*n* = 32)		TFAT			PT
		*p* = 0.05 *r* = 0.423			*p* = 0.05 *r* = 0.271
U16 (*n* = 40)		TFAT			
		*p* = 0.006 *r* = 0.505			
U17 (*n* = 23)		TFAA			
		*p* = 0.05 *r* = 0.509			

Abbreviations: KA, knee angulation; PA, pelvic angulation; PT, pelvic translation; SUM, total amount of investigated population; TFAA, total frontal anterior angulation; TFAT, total frontal anterior translation; TFDA, total frontal dorsal angulation; TFDT, total frontal dorsal translation; TSLT, total sagittal left translation.

The presence of recurrent injuries was additionally impacted by the total deviations observed in the frontal plane, namely TFDA, TFAT and TFAA, as well as the positioning of the pelvis, denoted as PT and PA. The strength of the correlation varied from moderate to strong, depending on the specific age cohort.

**Figure 6:**
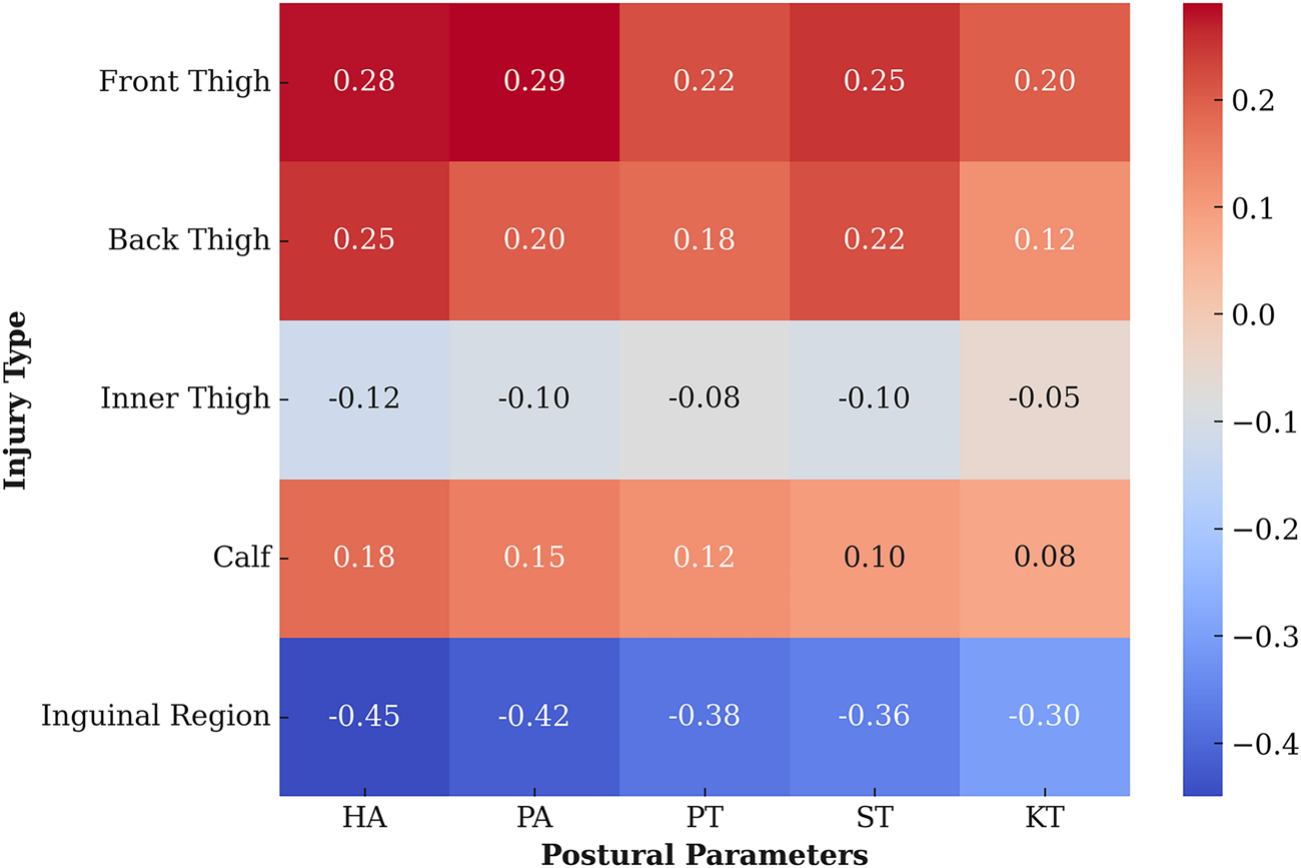
Heatmap of muscle injury types and associated postural parameters. Darkening of the colours indicates an increased risk of injury. HA, head angulation; KT, knee translation; PA, pelvis angulation; PT, pelvis translation; ST, shoulder girdle translation.

To clarify which type of muscle injury is more likely to occur when a specific postural parameter deviates, we applied discriminant analysis (Wilks's lambda 0.54). Considering standardised canonical discriminant coefficients, the variables with the highest absolute value are the most effective in discriminating between types of muscle injury. The HT, the ST, the PT, the KT and the HA played the most important role in classifying injuries. Inguinal injuries were the most correctly classified (57.1%), showing that these injuries have well‐defined postural correlates. The results are shown in Figure 6.

## DISCUSSION

The purpose of our research was to evaluate the association between poor posture and injury characteristics in male, youth football players. Our latest findings indicate that changes in postural parameters from the average in both sagittal and frontal planes may contribute to the development of various injuries. Given that adolescence is a crucial period for growth and subsequent changes in body biomechanics, we deemed it essential to compare the growth data of athletes with those of the general population. To achieve this, we compared the anthropometric results with reference data from the National Longitudinal Study of Child Growth, which provides national benchmarks for the reported means and percentiles of various dimensions measured from birth to age 18 [25]. The comparison revealed that within the study population of athletes, all age groups had higher sizes than the national average, particularly in the U13, U14 and U15 age groups in growth spurs.

Numerous studies [19, 37] have been conducted to examine injury rates in athletes within the age groups studied. These studies consistently indicate that a period of rapid growth has an impact on injury rates. Therefore, it can be concluded that the combination of rapid growth and biomechanical deficits associated with longevity increases the vulnerability to injury in immature organisms. This conclusion aligns with the findings of Wik's comprehensive review of 53 publications examining injury patterns in adolescent football players [53]. In terms of injury distribution, our results are very similar to findings by others. Our study detected that 84.4% of the 116 subjects reported injury. This aligns with other studies' results (65, 2%–91%) [7, 38]. Considering affected body parts, the injuries of lower extremities were dominant (91%) in our work, which is close to Frisch et al.'s (87%) and Deehan et al.'s (79%) results [11, 16]. Both of these studies examined the age‐and player‐related injuries in youth football. Our players suffered mostly muscle injuries (70.6%). A similar was observed by Hall et al. (58.5%) and Frisch et al. (63%), who also assessed the incidence of injury in young football players [16, 22]. The thigh was the most affected location (front of thigh 36%, Hamstring 44%) in our study, which number is slightly higher than in Wik's (25%) and Cezarino's (25.6%) research, looking for answers to the causes of injury patterns [7, 53]. Similarly, Towlson et al. reported a higher prevalence of gradual‐onset knee (Schlatter‐Osgood disease) and heel (severe disease) complaints among individuals in the U12–U14 age group, which is consistent with the present study [50]. Our research aligns with previous studies [7, 22], as we observed a rise in muscle injuries among 14‐ and 15‐year‐old athletes. The significance of this proportion is that youth football players could have a greater risk of injuries during or after rapid growth in stature. Previous research has consistently emphasised that maturity status plays a crucial role in shaping the structural body adaptations of young soccer players while also affecting their functional motoric potential [22, 35, 53]. In 54.1% of instances, athletes had to refrain from training for 1–3 weeks, while in 27.6% of cases, the absence extended to 4–6 weeks due to various injuries. These findings partially correspond to the existing literature [50], which indicates that injuries lasting over 4 weeks constitute 21%–26% of total cases, while shorter absences account for 31%–43% of cases.

### The relationship between postural parameters and injuries

The examination of posture among young football players with sagittal deviations has been extensively explored in various studies [4, 9, 50, 53]. Our findings indicate that both sagittal and frontal plane deviations are commonly observed in this sport and are closely associated with injury incidence. Using the PostureScreen application for our analysis, we discovered that postural parameters displayed the most significant deviations within the older age groups (U17–U19). The negative impact of long‐term participation in football on the postural abnormalities of players has been confirmed by our findings. These facts are consistent with the results of other studies, according to which posture is adapted to the form of movement of football [19, 45]. It is worth noting that the use of unilateral loads during football training can often lead to musculoskeletal disorders and further impact on the posture of athletes, potentially leading to pain and injuries. A recent study conducted by Theodorou et al. examined the effects of improper posture on specific motor skills in U15 and U17 soccer players [48]. Their results showed a clear association between poor posture and decreased performance on various physical tests, including the sit‐and‐reach test, the standing high jump test and isokinetic maximal effort test. These findings suggest that altered biomechanical conditions resulting from postural abnormalities may contribute to functional deficits, which in turn may indirectly increase the risk of certain injuries. Overall, however, a critical review of the findings suggests that postural changes adapted to football may also benefit sport performance, rather than solely increasing the risk of injury. In order to mitigate this risk, a research emphasised the importance of comprehensive assessment of athletes' posture as a tool for injury prevention [8, 26, 31].

Although many factors influence the development of poor posture, rapid growth in adolescent children is often behind poor posture. In addition, physicians usually recommend sport as a treatment option in addition to targeted strengthening to restore posture. However, in adolescent athletes, we need to consider the development of sport‐specific posture, which has been described in several studies [12, 17] as a possible cause of injuries and/or musculoskeletal complaints. During the growth spurt through adolescence, young football players are susceptible to traumatic and overuse injuries [12, 17] and biomechanical changes in the immature skeleton [36]. However, the relationship of injuries to biological maturity is often not well understood. From our approach, this means that attention should be paid to postural changes due to faster growth during adolescence on the one hand, and to postural changes caused by football on the other hand. Previous research [34, 49] on unilateral loading during football training acknowledges its effects on musculoskeletal adaptation. However, the conclusion that it directly results in postural deviations and subsequent injuries requires more rigorous longitudinal studies.

In our study, examining the relationship between injury classification and postural parameters, the differences observed in the sagittal and frontal planes affected each age groups variously. In particular, there was a positive correlation between the magnitude of the deviation and the probability of injury, indicating that as the magnitude of the deviation elevated, the risk of injury increased. As a novelty, we found that certain postural parameters can predict injury susceptibility, which are the parameters to watch out for during training. Inguinal region injuries show the strongest negative correlation with postural parameters, meaning this injury are associated with pelvic and shoulder girdle deviations. Front and back thigh injuries have moderate positive contributions from postural parameters, indicating that head and pelvis alterations influence them. Inner thigh injuries tend to show weaker (even slightly negative) correlations, suggesting they may depend on pelvic deviations. and injuries of the calf are likely to be behind knee deviations from the midline. The most important postural parameters related to the muscle injuries were pelvic tilt and displacement (PA, PT), head and shoulder position (HA, ST), knee and ankle displacements (KT).

A study by Snodgrass et al. on the extent of sagittal plane deviation reported a correlation between thoracic deviation and lower limb injury. However, in the same study, no association was found between lumbar lordosis deviation and injury incidence [45]. Ribeiro et al. also investigated the relationship between posture of competitive futsal players and lower limb injuries. They found an association between sagittal changes in the lumbar spine and injury incidence in 9–16‐year‐old players, reasoning that changes in posture may cause mechanical overload to musculoskeletal structures, predisposing the affected area to injury [40, 51]. Neither of these studies examined the extent of the discrepancy, only the fact of it. There is no literature reference on the examination of frontal plane deviations and their correlations with injuries. Our statistical prediction model classifies the inguinal region most correctly, so we could refine the model with additional biomechanical and training load parameters.

Contrary to our initial hypothesis based on the pooling of injury occurrence and postural parameter averages, we found more notable correlations within younger age groups than the older ones. It is worth noting that the outcomes for both postural and injury parameters were less favourable for the older age cohorts. In our analysis of preacademy and academy groupings, relatively weaker correlations indicating less significance were observed between posture and injury. This finding is likely due to the fact that postural parameters show greater variability across the population as a whole than within each age group. The relationship between injuries and poor posture has not been investigated before according to chronological age groups and academic classification.

There are no research investigating this question the only specific data found is that lordotic posture is more common in footballers and that the groyne is one of the most vulnerable areas. But there is no clear relationship. Our statistical prediction model classifies the inguinal region correctly, so we could refine the model with additional biomechanical and training load parameters.

We emphasise that the probability of recurrent injury is also influenced by postural factors in our findings. It is important to know that the probability of recurrent injury is influenced by postural factors. Differences in frontal plane, such as TFAT, TFDT, TSRT, TSLT, TFAA, TFDA, TSRA and TSLA, as well as pelvic positioning, may have a significant impact on the recurrence of injuries across various age groups. Our study indicates that the risk of injury can be attributed to both individual variation in specific body parts and the general variation in ‘total’ parameters, from prepeak to older age groups, a worsening trend at the expense of older age groups due to biomechanical changes caused by rapid growth. It is worth emphasising, however, that our research is not exhaustive, as we have not encountered any studies that investigate the relationship between the extent of postural deviation and different types of injuries or recurrence of injuries. In the literature that can be found, repeated injuries are not indicated, but previous ones [48]. Given the paucity of research in this area, it is necessary to investigate the causes of repetitive injuries to obtain more reliable results. In the context of football injuries, we highlight the importance of addressing frontal plane postural deviations, alongside sagittal deviations, to reduce the risk of injuries.

The assessment and analysis of posture in athletes is an essential component of patient assessment in physiotherapy, serving as a valuable resource for both individual and team sports. Through this process, we are able to identify factors that contribute to the risk of injury and develop targeted exercise regimens for preventative measures. To aid this analysis, we use the PostureScreen application, which offers a convenient and efficient means of assessment. Our experience has shown that this application can be readily implemented at training sites, providing results that closely align with those obtained through more expensive and specialised laboratory posture analysis methods. However, a more balanced approach, incorporating neuromuscular screening, movement quality assessment and workload monitoring, may provide a more comprehensive framework for injury prevention.

## CONCLUSIONS

In our study, we observed a significant incidence of poor posture in athletes, which was associated with a strikingly high injury rate compared to existing literature. Through our research, we have provided a comprehensive analysis of the correlation between postural abnormalities and the frequency of injuries, thereby offering valuable insights to enhance the precision and effectiveness of practical sports training. Our research demonstrated an association between poor posture, including both frontal and sagittal deviations, and the injury incidence among adolescent football players.

### Limitations

The limitation of our study was that we have not recorded any data with regards to the participants' daily or habitual activities other than soccer training. Due to the limited sample size within each age group, it is important to extend the sample to a larger population.

## AUTHOR CONTRIBUTIONS

All authors have approved the manuscript and agree with its submission.

## CONFLICT OF INTEREST STATEMENT

The authors declare no conflicts of interest.

## ETHICS STATEMENT

The protocol was approved by the Regional and Institutional Committee of Science and Research Ethics (permit number: 7939‐PTE 2019, date of approval: 08/06/2019). All participants read and signed an informed consent.

## Data Availability

The data presented in this study are openly available in FigShare at https://doi.org/10.6084/m9.figshare.25567845.
